# A hybrid Ornstein–Uhlenbeck–Branching framework unifies microbial and pediatric tumor evolution

**DOI:** 10.3389/fonc.2026.1727973

**Published:** 2026-02-13

**Authors:** Seung-Hwan Kim

**Affiliations:** Department of Biology, Fisher College, Boston, MA, United States

**Keywords:** branching process, clonal dynamics, microbial evolution, Ornstein–Uhlenbeck process, pediatric cancer evolution, precision oncology

## Abstract

**Introduction:**

Pediatric tumors can relapse despite low mutation burdens, suggesting hybrid evolutionary dynamics shaped by stochastic variability and stabilizing forces. We develop a hybrid Ornstein–Uhlenbeck (OU)–Branching framework that couples mean-reverting stochastic trait dynamics with demographic birth–death processes to model lineage diversification under effective stabilizing constraints.

**Methods:**

Using Escherichia coli long-term evolution experiment (LTEE) lineages (WT, priA, recG), we parameterized the equilibrium mean (μ), mean-reversion strength (θ), and diffusion scale (σ) on the log10 mutation-frequency axis via replicate-grouped likelihood inference. We performed forward simulations for predictive envelopes, uncertainty quantification, phase-plane dynamics, and OU–Branching lineage networks. We also ran illustrative in silico therapy simulations under fixed OU parameters with exposure-modulated birth/death rates.

**Results:**

The fitted model recapitulated lineage-specific mutation dynamics and branching architectures. priA exhibited elevated stochastic dispersion and drift-prone behavior consistent with a high-plasticity regime, whereas recG showed constrained diversification and increased lineage turnover consistent with a collapse-prone regime. Illustrative therapy simulations generated oscillatory trait trajectories, suppression–rebound population dynamics, clonal pruning, and extinction-versus-persistence regimes.

**Discussion:**

Although Y is directly observed as log10 mutation frequency in LTEE, in tumors Y can represent a longitudinally measurable phenotypic state (e.g., drug-tolerance scores from single-cell data, MRD/VAF-derived burden proxies, or pathway activity states). The balance between stabilizing strength (θ) and stochastic variability (σ) provides a quantitative axis governing plasticity and persistence, motivating future calibration to clinical longitudinal data for evolution-aware, patient-specific modeling.

## Introduction

Pediatric cancers exhibit low mutation burdens and relatively simple karyotypes yet display aggressive behavior and complex evolutionary patterns (1–5). This paradox suggests that progression is driven less by continuous mutational accumulation and more by stochastic clonal selection and stabilizing forces operating within constrained phenotypic spaces (6–9). Understanding how variability, selection, and turnover interact to shape these dynamics remains a key challenge in precision oncology.

Traditional evolutionary models emphasize either deterministic selection or stochastic drift; however, pediatric cancers likely evolve through a hybrid regime in which stabilizing selection preserves essential traits while random fluctuations enable clonal diversification and adaptive escape (6, 7, 10, 11). Capturing this balance requires a framework that links phenotypic reversion toward equilibrium with probabilistic clonal branching.

**Figure 1:**
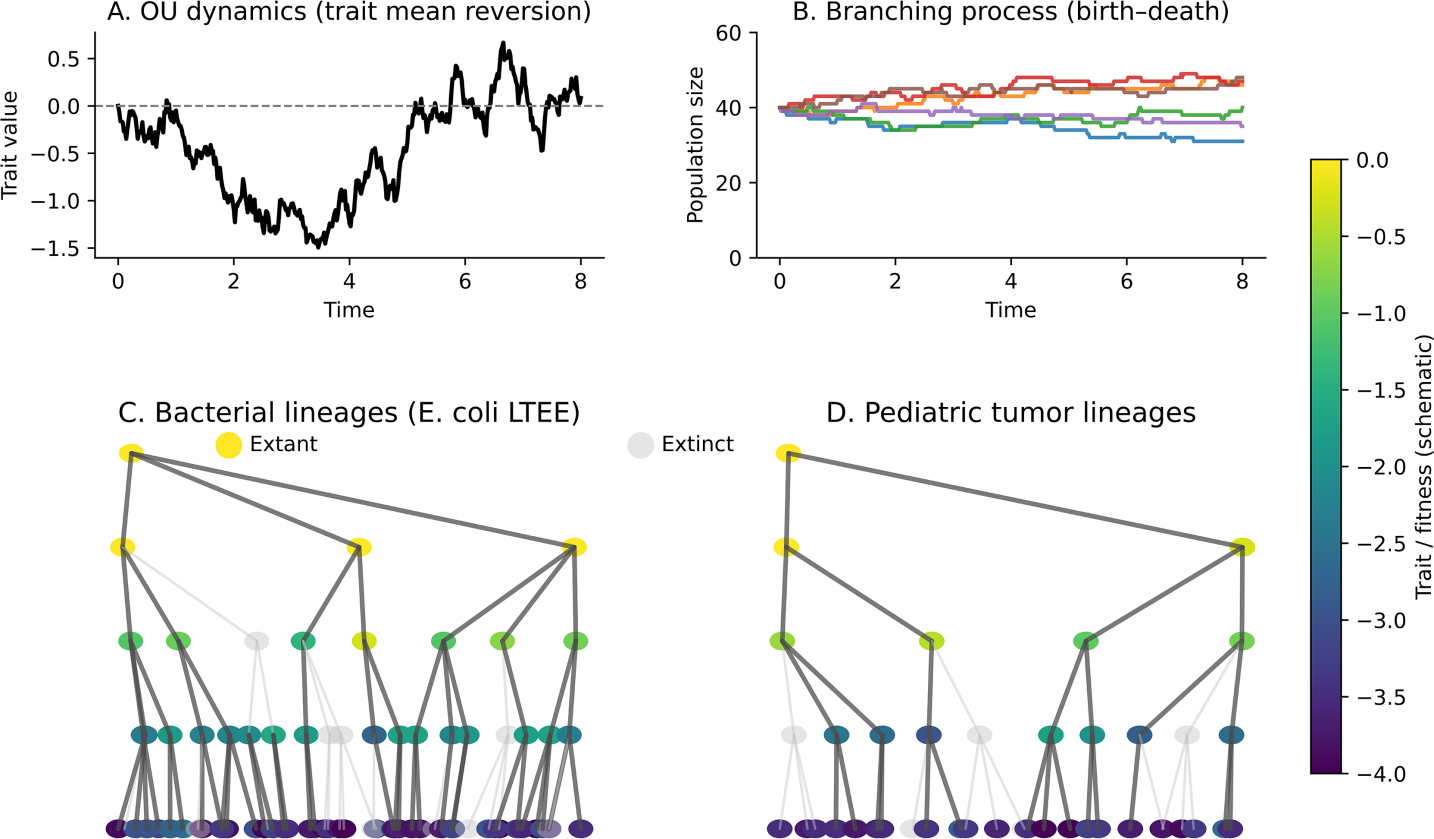
Linking OU traits to branching lineage dynamics. **(A)** Ornstein–Uhlenbeck (OU) dynamics illustrating mean reversion toward an equilibrium mean *μ* under stochastic diffusion *σ* and stabilizing strength *θ*. **(B)** Birth–death branching process showing stochastic clonal growth and extinction. **(C)** Schematic of *E*. *coli* LTEE lineages modeled under the OU–Branching framework. **(D)** Analogous schematic of pediatric tumor lineages showing diversification and extinction driven by OU-governed phenotypic evolution and branching proliferation.

In this study, a hybrid Ornstein–Uhlenbeck (OU) and branching-process model was developed to unify stochastic trait evolution with demographic birth–death dynamics (12, 13). The OU component describes mean-reverting phenotypic fluctuations around a lineage mean (*μ*) on the log_10_ mutation-frequency scale, whereas the branching component captures random proliferation and extinction events that generate evolving lineage networks. Together, these coupled processes simulate how stabilizing selection and stochastic diversification jointly shape clonal evolution (**Figure 1**).

As an experimental analog, the *Escherichia coli* long-term evolution experiment (LTEE) recapitulates general evolutionary phenomena relevant to tumors—mutation accumulation, clonal interference, and adaptive constraint—over measurable timescales (**Supplementary Figures S1–S2**) (14). A comparison of wild-type, *priA* (replication restart-deficient), and *recG* (replication fork repair-deficient) lineages quantifies lineage-specific mutation rates, phenotypic variance, and stabilizing selection strength (15–19). The *priA* mutant exhibits hypermutable, drift-prone behavior analogous to mutator subclones in relapsed pediatric cancers, whereas *recG* shows dynamics consistent with a collapse-prone regime under replication stress.

**Figure 2:**
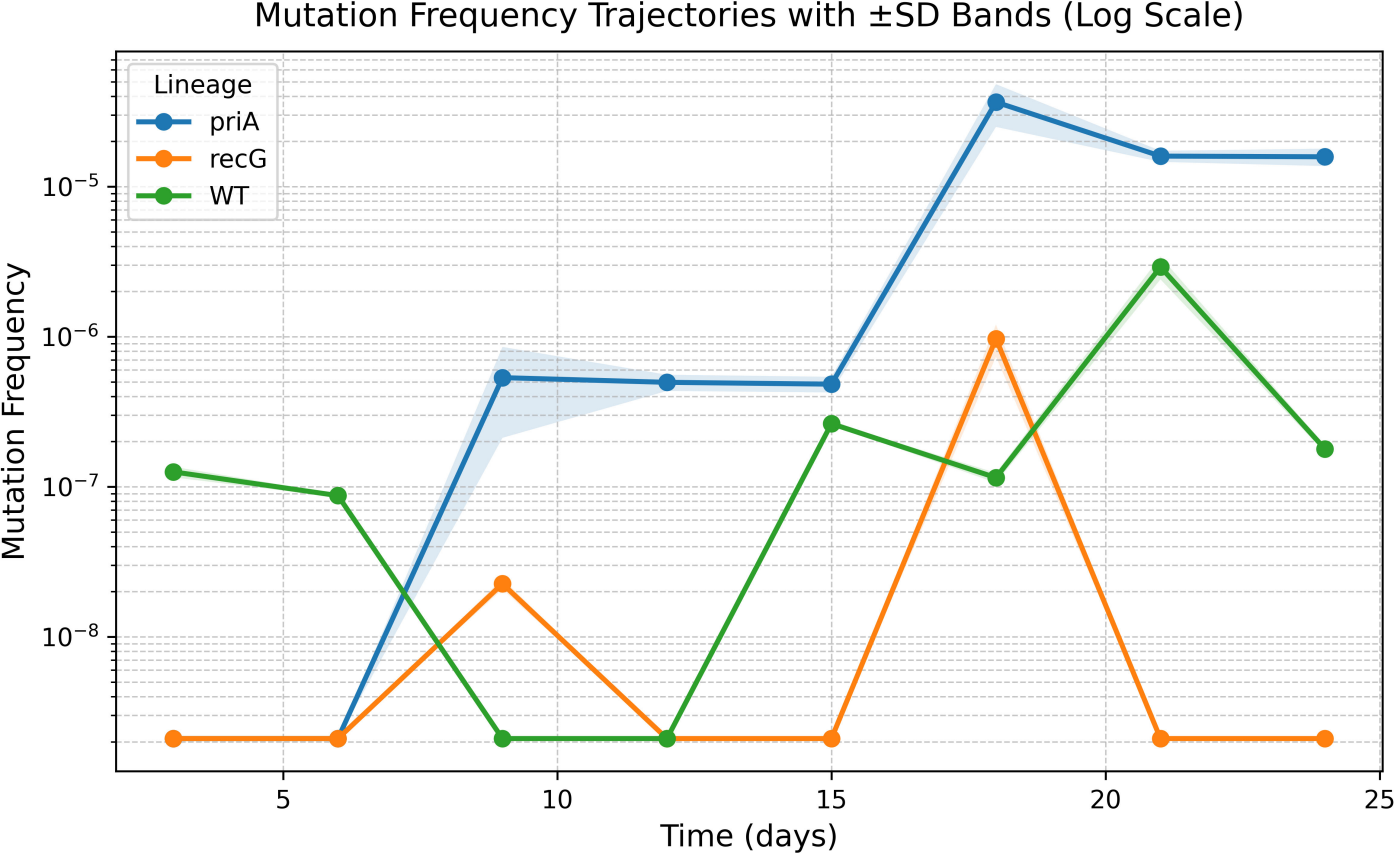
Mutation-frequency trajectories and OU model fitting. Log-scale mutation-frequency trajectories in *E. coli* LTEE lineages (WT, *priA, recG*). Lines show replicate means ± SD. OU model fitting supports lineage-specific dynamics: *priA* exhibits an elevated equilibrium level (higher 10^*μ*) and greater stochastic dispersion, whereas WT and *recG* show more constrained trajectories. A likelihood-ratio test rejects a shared-parameter model (2ΔlogL = 33.18, df  = 6, *p* < 9.7 x 10^-6^), supporting lineage-specific OU parameters (**Table 2**).

**Figure 3:**
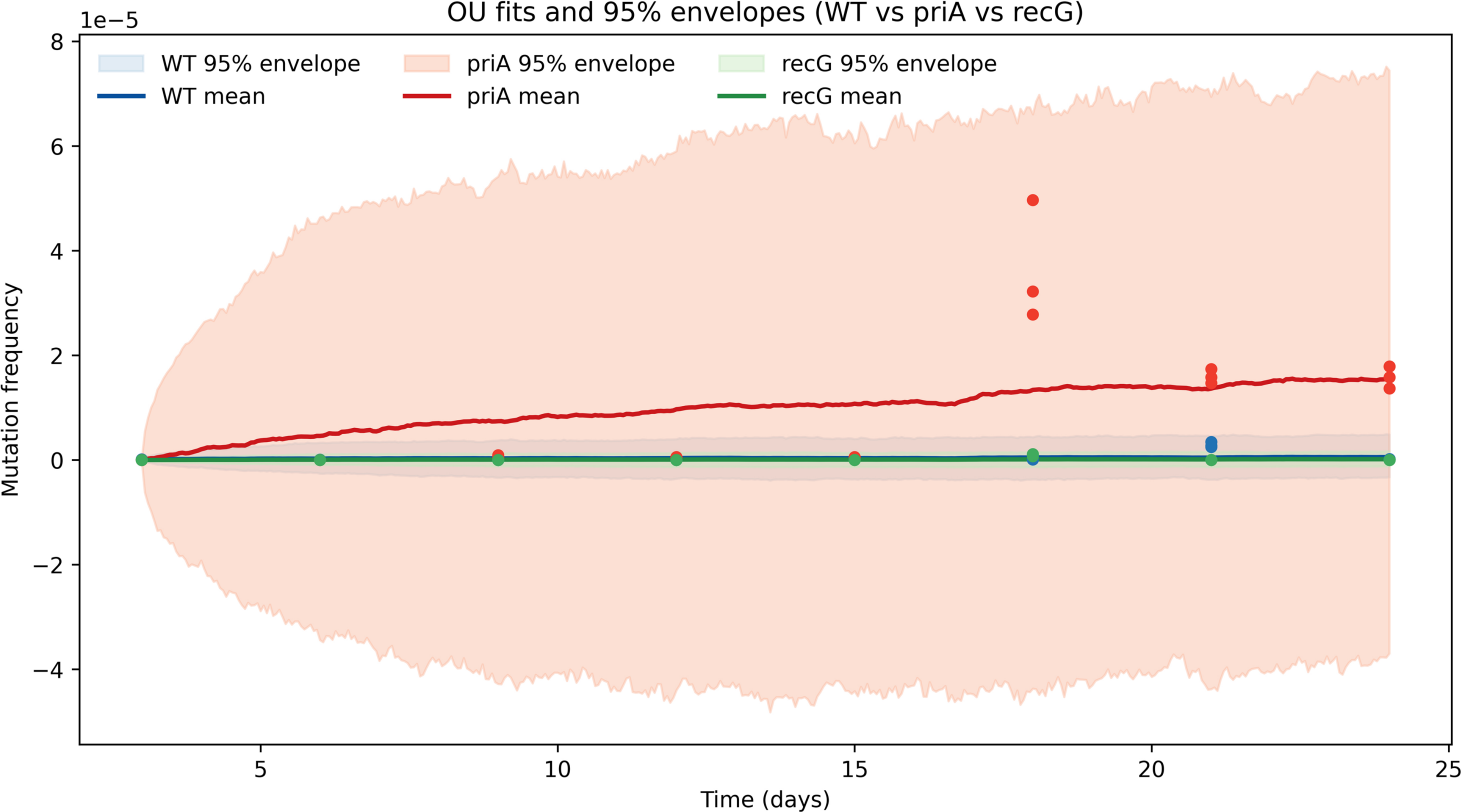
OU model fits and 95% envelopes for mutation dynamics. OU model fits for *E. coli* lineages (WT, *priA, recG*) with 95% predictive envelopes for mutation dynamics. Solid lines show fitted means; shaded areas indicate predictive intervals from forward simulation under fitted parameters. *priA* exhibits a higher mean mutation frequency and greater variability, whereas WT and *recG* remain within narrower envelopes. The OU-derived envelopes capture lineage-specific evolutionary constraints.

**Figure 4:**
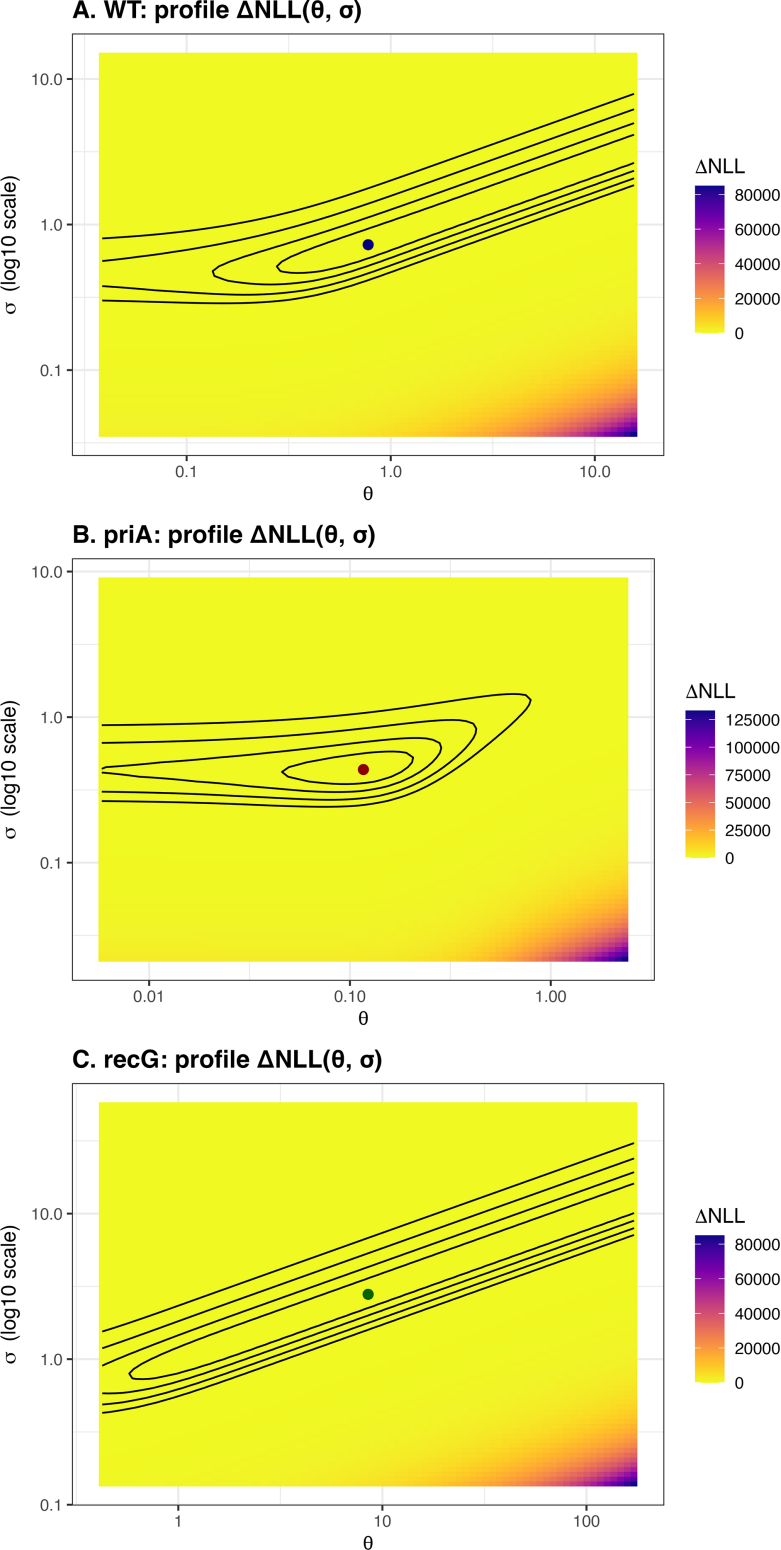
Profile-likelihood surfaces of OU parameters (*θ*, *σ*). **(A-C)** Profile-likelihood heatmaps for *E. coli* lineages (WT, *priA, recG*) showing ΔNLL relative to the lineage-specific minimum as a function of (*θ, σ*) with *μ* fixed at its MLE. Black contours denote fixed ΔNLL levels. Points mark the MLEs for each lineage. *priA* displays a broader, asymmetric basin consistent with strong *θ*–*σ* coupling, whereas WT and *recG* show more sharply localized optima.

**Figure 5:**
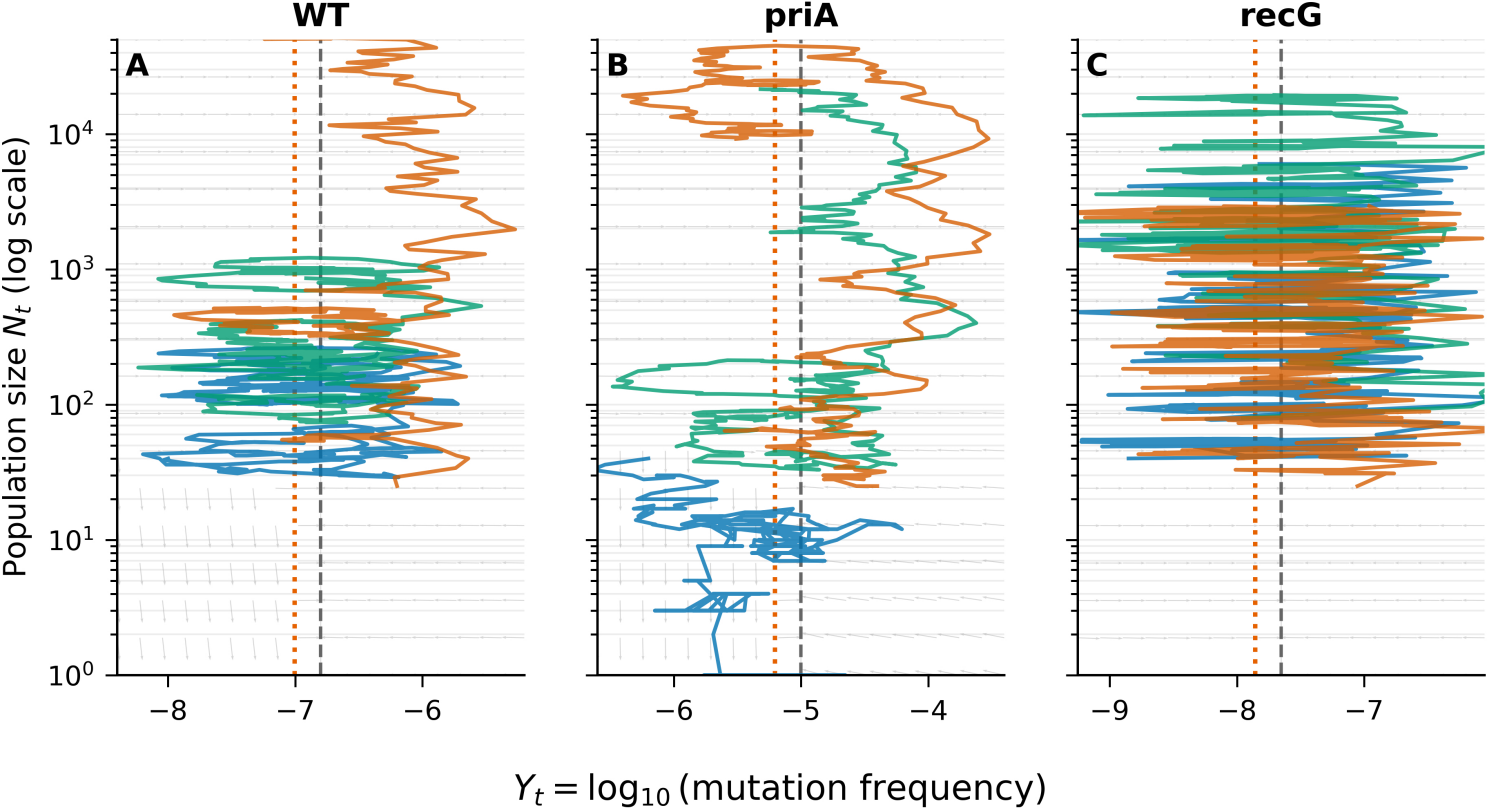
Phase-plane trajectories of the hybrid OU–Branching model. **(A–C)** Phase-plane plots coupling phenotypic evolution ( $Y_{t}$ = log_10_ mutation frequency) and population dynamics ( $N_{t}$, log scale) for WT, *priA*, and *recG*, simulated using lineage-specific replicate-grouped OU parameters (**Table 1**). Gray arrows show the deterministic vector field defined by *dY*/*dt* = *θ*(*μ* − *Y*) and *dN*/*dt* = (*λ*(*Y*) − *δ*(*Y*))*N*. The gray dashed vertical line marks the lineage mean phenotype *μ*, and the orange dotted vertical line marks the initial phenotype $Y_{0}$; the demographic nullcline occurs where *λ*(*Y*) = *δ*(*Y*). Colored curves show representative stochastic OU–Branching trajectories, illustrating lineage-specific differences in constraint, stochastic dispersion, and demographic response. How to read this figure: first use the arrows to interpret the expected (deterministic) drift in (*Y*, *N*), then compare how stochastic trajectories fluctuate around *μ* and across the demographic nullcline to reveal differences in persistence and collapse tendencies across lineages.

**Figure 6:**
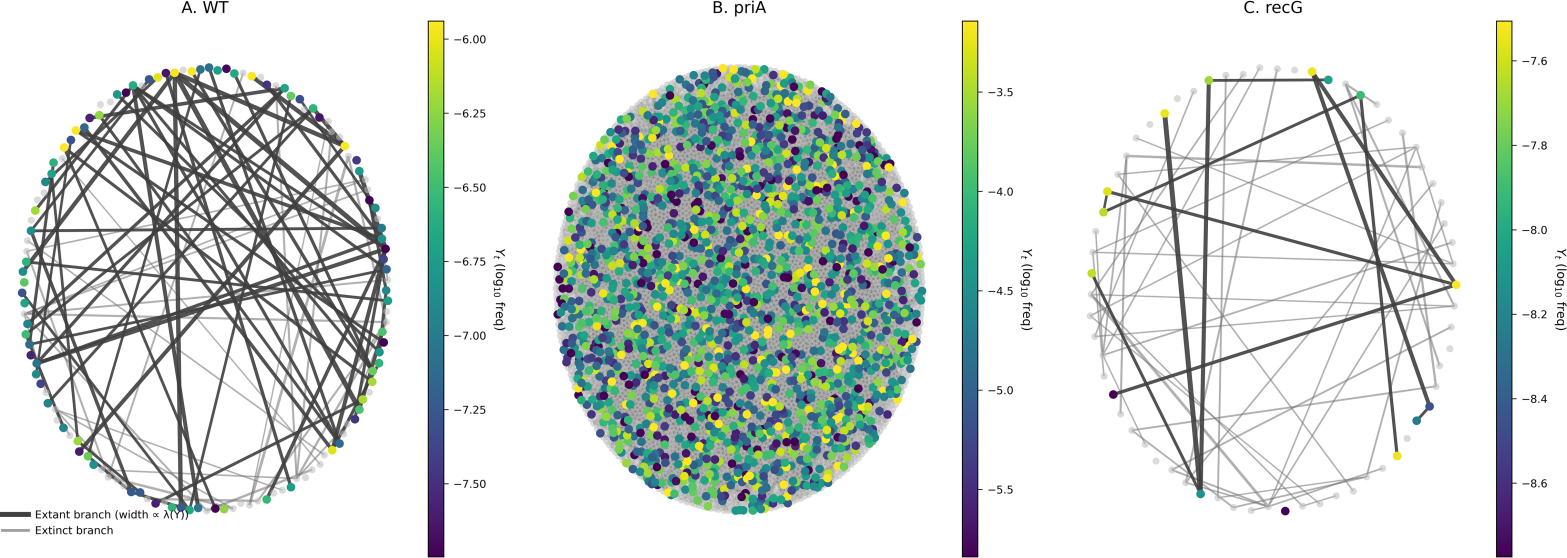
Hybrid OU–Branching lineage snapshots across *E. coli* lineages. **(A–C)** Lineage graphs for WT, *priA*, and *recG* were generated under phenotype-coupled birth–death dynamics using lineage-specific replicate-grouped OU parameters (**Table 1**) on the log_10_ mutation-frequency scale. Extant clones are colored by phenotype $Y_{t}$ = log_10_(mutation frequency) using a viridis scale, while extinct clones are shown in gray. Edge thickness is proportional to the instantaneous division rate *λ*(*Y*); darker edges denote extant branches, and lighter edges denote extinct branches. For visualization, the *priA* panel was downsampled to a maximum of 2000 extant nodes without altering the underlying simulation. How to read this figure: node color encodes phenotype, gray nodes indicate extinct lineages, and thicker/darker edges highlight branches with higher division rates and extant descendants.

**Figure 7:**
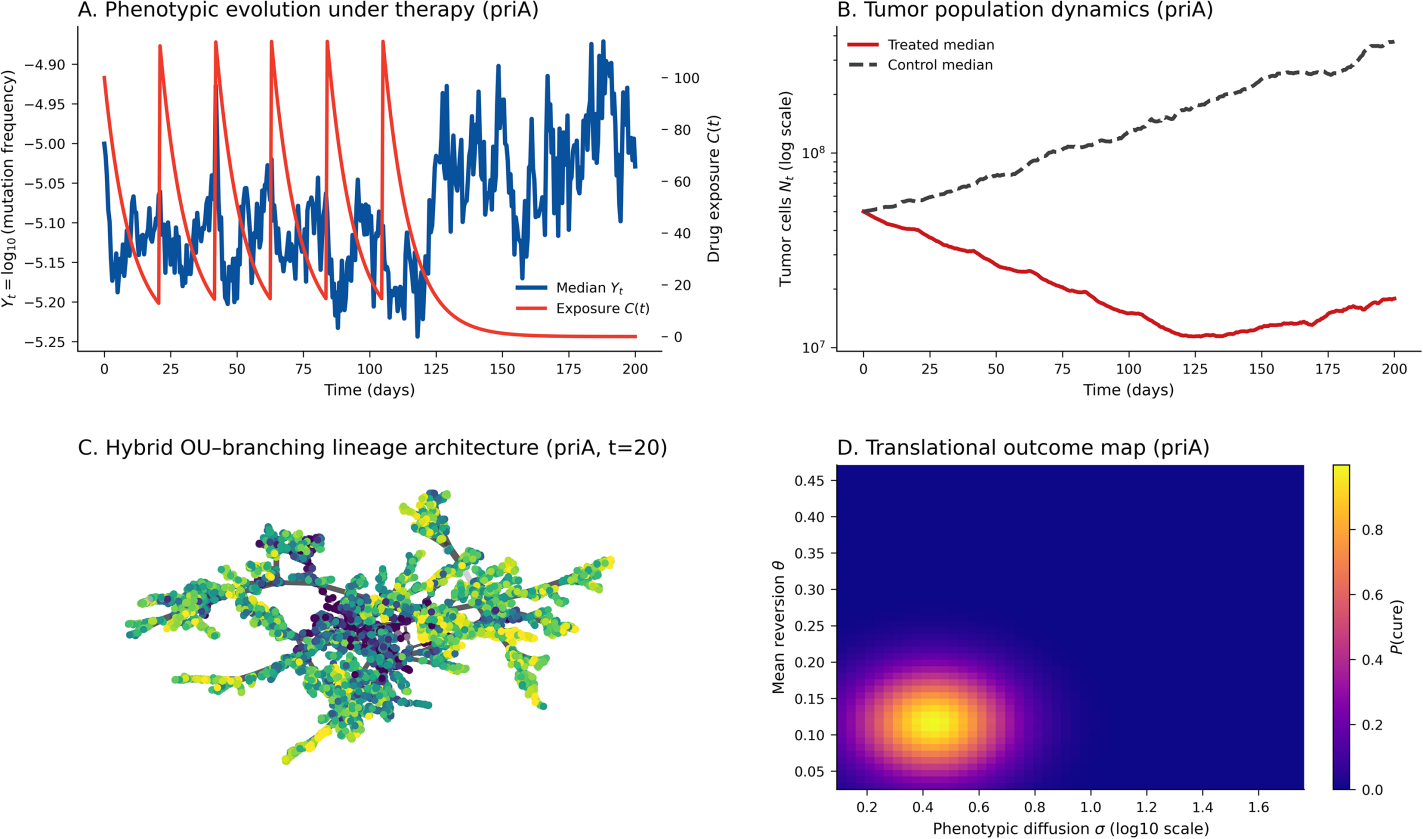
Translational application of the hybrid OU–Branching framework (*priA* profile). **(A)** Phenotypic evolution under cyclic exposure showing the median trajectory of $Y_{t}$ = log_10_(mutation frequency) (blue) alongside drug exposure *C(t)* (red) across six 21-day cycles. **(B)** Simulated population dynamics on a log scale comparing treated (red) versus untreated control (gray dashed) median trajectories. **(C)** Hybrid OU–Branching lineage architecture at the shared snapshot time *t*  = 20, with clones colored by phenotype and edges scaled by the instantaneous division rate *λ*( $Y_{t}$, *C(t)*). **(D)** Illustrative outcome map showing how *P(cure)* varies with phenotypic diffusion *σ* (log_10_ scale) and mean reversion *θ*. Panels **(A–C)** use the *priA* profile parameters inferred from replicate-grouped OU fits (**Table 1**). How to read this figure: exposure *C(t)* specifies the imposed schedule, while trajectories and lineage graphs are forward simulations under fixed OU parameters; the panels are intended to be hypothesis-generating and not predictive of clinical efficacy or optimal scheduling.

This framework links mutation-frequency dynamics, parameter inference, and lineage topology (**Figures 1**–**6**) to a unified view of evolutionary equilibria. Its extension to therapy simulations (**Figure 7**) provides an illustrative forward-simulation testbed for how cyclic exposure can reshape trait dynamics and clonal persistence. These simulations are hypothesis-generating and motivate future calibration on longitudinal clinical data.

Overall, this study establishes a multiscale OU–Branching framework that links microbial evolutionary dynamics to tumor-relevant principles of constrained adaptation, stochastic diversification, and clonal turnover. By integrating stabilizing selection (*θ*) and stochastic variability (*σ*) with phenotype-coupled birth–death demography, the model yields interpretable regime behavior—stabilized, hypermutable/plastic, and collapse-prone—supported by likelihood-based inference, uncertainty quantification, and lineage-architecture simulations (**Figures 1**–**6**). Importantly, the same formalism is designed to transfer to pediatric tumors by defining $Y_{t}$ as any longitudinally measurable state variable, such as drug-tolerance or stress-response programs inferred from single-cell data, Minimal Residual Disease (MRD)–based burden proxies, or clonal fraction trajectories derived from time-series sequencing. In that setting, patient- or cohort-specific OU parameters (*μ*, *θ*, *σ*) can be estimated from repeated measurements using the same likelihood-based framework. Finally, the therapy simulations (**Figure 7**) are presented as an illustrative forward-simulation testbed showing how cyclic treatment can reshape trait dynamics and clonal persistence; this provides a practical roadmap for future calibration on clinical time-series data to support precision, evolution-aware pediatric oncology.

## Methods

### Estimation of lineage-specific parameters

Mutation-frequency data from *E. coli* LTEE populations (WT, *priA*, *recG*) (**Supplementary Figures S1–S2**; **Supplementary Table S1**) were preprocessed on a per-lineage basis to enable log-scale modeling. Let $x_{r,t}\geq 0$ denote the observed mutation frequency for replicate *r* at time *t*. In this microbial application, $Y_{t}$ is directly observed as $y_{r,t}$, the log_10_ floored mutation frequency for replicate *r* at time *t*. The log_10_ mutation frequency corresponds to that measured for replicate *r*. To avoid undefined log_10_(0), zeros were replaced using a lineage-specific detection-floor constant

$\epsilon =0.5\;\times \;min\{x_{r,t}\;:x_{r,t}>0\}$,

and we defined the floored frequency

$x_{r,t}^{*}=max(x_{r,t},\epsilon )$.

The analyzed trait was

$y_{r,t}={\text{log}}_{10}(x_{r,t}^{*})$.

### Rationale for OU dynamics

We model the log_10_ mutation-frequency trajectory $y_{r,t}$ using an OU process because the observed time series exhibit bounded stochastic fluctuations around lineage-specific levels rather than unbounded random-walk drift, consistent with mean-reverting dynamics under stabilizing constraints. The OU model also yields a closed-form transition density under irregular sampling intervals, enabling likelihood-based inference and reproducible uncertainty quantification from replicate trajectories.

Lineage-specific Ornstein–Uhlenbeck (OU) parameters *μ* (equilibrium mean), *θ* (mean-reversion rate), and *σ* (diffusion scale) were estimated by maximizing the exact OU transition likelihood, using a replicate-grouped objective (sum of negative log-likelihood across replicate trajectories). Optimization used bounded L-BFGS-B with constraints *θ* > 0 and *σ* > 0, with multiple random restarts to mitigate local optima. Lineage-specific fits were compared against a shared-parameter null using a likelihood-ratio test (LRT; df = 6). Parameter stability and identifiability were evaluated by curvature near the optimum and by profile-likelihood surfaces in (*θ, σ*) space.

### Simulation of Ornstein–Uhlenbeck and branching dynamics

All simulations were based on the OU stochastic differential equation:


$$dY_{t}=\theta (\mu -Y_{t})dt+\sigma dW_{t}$$

where $Y_{t}$ is the phenotypic trait, *μ* the equilibrium mean, *θ* the mean-reversion rate, *σ* the diffusion scale (volatility; variance is *σ^2^*), and $W_{t}$ a Wiener process (20–23). OU trajectories were simulated using the exact discrete-time solution over eight arbitrary time units (illustrative schematic for **Figure 1**; not used for parameter inference) with parameters *θ* = 0.8, *σ* = 0.3, and *dt* = 0.01. In this framework, *θ* is interpreted as an effective stabilizing strength acting on the modeled trait $Y_{t}$. Depending on the biological instantiation, *θ* may reflect direct stabilizing selection on $Y_{t}$ or an emergent mean-reverting constraint arising from population-genetic and cellular limits (e.g., repair capacity, trade-offs, clonal interference, or ecological feedback). Distinguishing these mechanisms would require additional orthogonal measurements beyond the scope of the present study.

Branching processes followed a stochastic birth–death model with per-capita rates *b* = 0.05 and *d* = 0.04, initialized with 40 individuals per replicate. Hybrid OU–Branching lineage schematics were implemented in Python (NumPy + NetworkX + Matplotlib), where each clone inherited its parental trait plus OU noise, and survival probability scaled with trait-dependent fitness. Extant nodes were colored by phenotype (viridis), and extinct nodes were shown in gray and identified at the snapshot time (*t* = 20; **Figures 1**, **6**, **7**).

### Model validation and forward simulations

OU model validation used the exact transition density and forward simulations (10,000 replicates per lineage) under fitted parameters. Predictive envelopes were generated from the 2.5th to 97.5th percentiles at each time point, representing 95% predictive intervals. Observed mutation frequencies were overlaid with fitted mean trajectories. Parameter stability was corroborated by the curvature of the log-likelihood surface near the optimum (**Figure 3**) and the profile-likelihood geometry in (*θ, σ*) (**Figure 4**).

### Bootstrap uncertainty quantification of OU parameters

To quantify estimation uncertainty for lineage-specific OU parameters, we performed a nonparametric bootstrap over biological replicates within each lineage (WT, *priA, recG*). For each bootstrap iteration (B = 2000), replicate trajectories were sampled with replacement, and OU parameters (*μ, θ, σ*) were refit by maximum likelihood using the same replicate-grouped transition likelihood. Percentile-based 95% intervals were computed from the bootstrap distributions for *μ, θ*, and *σ*. To characterize parameter coupling, we summarized the correlation between log(*θ*) and log(*σ*) across bootstrap refits (**Table 1**).

**Table 1:** OU parameter estimates with replicate-grouped bootstrap 95% intervals.

Lineage	*μ* (log_10_) [95% CI]	10^*μ* (freq) [95% CI]	*Θ* [95% CI]	*σ* [95% CI]	*σ²*/(2*θ*) (log_10_ var) [95% interval]
WT	-6.799 [-6.809, -6.785]	1.588e-07 [1.554e-07, 1.640e-07]	0.775 [0.736, 0.808]	0.727 [0.701, 0.763]	0.341 [0.319, 0.368]
*priA*	-5.000 [-5.168, -4.702]	9.991e-06 [6.789e-06, 1.985e-05]	0.117 [0.084, 0.162]	0.436 [0.391, 0.494]	0.817 [0.753, 0.909]
*recG*	-7.652 [-7.675, -7.638]	2.229e-08 [2.112e-08, 2.303e-08]	8.516 [6.521, 13.785]	2.789 [2.394, 3.549]	0.457 [0.396, 0.488]

Parameters are estimated on log_10_-transformed mutation-frequency trajectories $Y={\text{log}}_{10}(x_{adj})$, where $x_{adj}=x$ for x >0, and zeros were replaced within each lineage by ϵ=0.5 × min(x>0). Maximum-likelihood estimates (MLEs) were obtained using the exact OU transition density with the negative log-likelihood summed across replicate trajectories (replicate-grouped likelihood; *θ, σ* > 0). Uncertainty intervals are nonparametric replicate bootstraps (B = 2000), resampling replicate trajectories with replacement and refitting the model; reported intervals are the 2.5th–97.5th percentiles. The equilibrium mutation frequency on the original scale is 10^*μ*, and the stationary variance on the log_10_ scale is *σ²*/(2*θ*); both are summarized by percentile intervals computed directly from bootstrap samples.

### Profile-likelihood surface analysis

Profile-likelihood surfaces of *θ* and *σ* were computed in R (v4.3.0). For each lineage, the maximum-likelihood estimate (MLE) of μ was fixed, and log-spaced grids of *θ* and *σ* were explored within ±3 log units around the optimum.

### Exact OU transition and NLL

Let $y_{i}$ denote the observed trait at time $t_{i}$ and $\text{Δ}t_{i}=t_{i}-t_{i-1}$. Under the OU transition,

$y_{i}\;|\;y_{i-1}\;\sim \;N(m_{i},v_{i}),\;$where

$m_{i}=\mu +(y_{i-1}-\mu )e^{-\theta \text{Δ}t_{i}}$,

$v_{i}=\frac{\sigma^{2}}{2\theta }(1-e^{-2\theta \text{Δ}t_{i}})$.

The negative log-likelihood is

$\text{NLL}(\mu ,\theta ,\sigma )=\frac{1}{2}\;\underset{i}{\sum }[\text{log}(2\pi v_{i})+\frac{{\left(y_{i}-m_{i}\right)}^{2}}{v_{i}}]$.

Because the OU transition density is closed-form under irregular sampling intervals, likelihood-based estimation (MLE/NLL minimization) provides a standard, reproducible inference approach for longitudinal trajectories.

Contours at fixed ΔNLL levels were visualized using ggplot2 and viridis (**Figure 4**). Fit summaries (NLL, AIC, and ΔAIC) are reported for reference using AIC = 2k + 2×NLL (k = 3 parameters) (**Table 2**).

**Table 2:** Model comparison between a shared-parameter OU model and a lineage-specific OU model.

Model	k	NLL	AIC	ΔAIC	LRT	df	*p*
Shared-parameter OU (null)	3	77.0154	160.0308	21.1838	–	–	–
Lineage-specific OU (alt)	9	60.4235	138.8469	0	33.1838	6	9.7 x 10^-6^

Negative log-likelihood (NLL) and AIC (AIC = 2k + 2×NLL) are reported for each model fit to the same dataset (WT + *priA* + *recG*). ΔAIC is relative to the smallest AIC in this table. The likelihood-ratio statistic is LRT = 2(NLL_null − NLL_alt) = 2ΔlogL, with df = k_alt − k_null (= 6). We report AIC (rather than BIC) because longitudinal trajectories can exhibit temporal correlation, which reduces the effective sample size, and AIC is commonly used to compare predictive fit among candidate likelihood models in such settings.

### Formal quantitative trajectory separation metrics

To complement predictive envelopes, we quantified differences between lineage trajectories using model-implied predictive distributions. For each lineage, the OU predictive mean m(t) and variance v(t) were computed analytically from fitted parameters. Pairwise comparisons between lineages A and B were summarized across time points by:

- mean absolute separation of predictive means $D_{mean}=\;mean_{t}|\;m_{A}(t)-\;m_{B}(t)|$;
- the averaged 2-Wasserstein distance between the 1D Gaussian predictive distributions $N(m_{A}(t),\;v_{A}(t))$ $N(m_{B}(t),\;v_{B}(t))$, $W_{2}(N(m_{A},v_{A}),\;N(m_{B},v_{B}))=\;\sqrt{{\left(m_{A}-m_{B}\right)}^{2}+{\left(\sqrt{v_{A}}-\sqrt{v_{B}}\right)}^{2}};\;$and
- a dominance probability $P(A>B)=\;mean_{t}[\text{Φ}(\frac{m_{A}(t)-m_{B}(t)}{\sqrt{v_{A}(t)+v_{B}(t)}})]$. Uncertainty intervals for these trajectory metrics were obtained by recomputing the metrics across paired bootstrap draws from each lineage’s (*μ, θ, σ*) distribution (**Table 3**).

**Table 3:** Pairwise trajectory separation metrics with bootstrap 95% intervals.

Pair (A vs B)	D_mean (log10) [95% CI]	Approx. fold (10^D_mean) [95% CI]	D_W2 (log10) [95% CI]	P(A > B) [95% CI]
*priA* vs *recG*	1.944 [1.912, 1.987]	87.818 [81.712, 96.971]	1.950 [1.918, 1.993]	0.968 [0.963, 0.973]
*priA* vs WT	1.045 [1.018, 1.091]	11.103 [10.422, 12.338]	1.069 [1.041, 1.113]	0.876 [0.865, 0.891]
*recG* vs WT	0.898 [0.881, 0.917]	7.909 [7.595, 8.267]	0.903 [0.889, 0.919]	0.150 [0.136, 0.157]

Metrics were computed from OU predictive distributions on the log_10_ mutation-frequency scale, propagating uncertainty using replicate-grouped bootstrap draws of (*μ*, *θ*, *σ*) (**Table 1**; B = 2000). For each bootstrap draw, trajectories were predicted on the time grid *t* = [0, 3, 6, 9, 12, 15, 18, 21] (relative to the first observation), and pairwise separation was summarized by D_mean and D_W2 (both non-negative magnitude metrics). Approximate fold-change is computed as 10^D_mean within each bootstrap draw before taking quantiles. P(A > B) denotes the probability that lineage A exceeds lineage B; values below 0.5 indicate that B tends to be larger. Reported values are the bootstrap median (q50) with 95% intervals [q025, q975].

### Phase-plane simulation of coupled dynamics

Phase-plane diagrams combined OU phenotypic evolution with phenotype-dependent birth–death dynamics. Deterministic vector fields visualized the skeleton *dY*/*dt* = *θ*(*μ* − *Y*) and *dN*/*dt* = (*λ*(*Y*) − *δ*(*Y*))*N*, where *λ* and *δ* depend on deviation from the lineage mean (*Y* − *μ*). Stochastic trajectories were simulated with exact discrete-time OU updates (*Δt* = 0.05) and Poisson birth/death events, and rendered with log-scaled *N* (**Figure 5**). For constant-rate simulations, we use *b*, *d*; for phenotype-coupled simulations, we use *λ*(*Y*), *δ*(*Y)* with *b* = *λ_0_* and *d = δ_0_* as baseline rates.

### Network construction of hybrid OU–Branching lineages

Lineage networks were generated using lineage-specific OU parameters (**Table 1**) and phenotype-coupled birth–death dynamics. At each time step, extant clones updated *Y* via the exact OU transition and produced offspring with division rate $\lambda (Y)=\;\lambda_{0}\cdot exp(\alpha \cdot (Y-\mu ))$ and death rate $\delta (Y)=\;\delta_{0}\cdot exp(-\beta \cdot (Y-\mu ))$, with rates clipped for numerical stability. Simulations were run for T = 40 time units, with extant and extinct clones identified at the snapshot (*t* = 20) for all lineages. Extinct clones were shown in gray, and extant clones were colored by phenotype using viridis; for visualization, the *priA* panel was downsampled to a maximum of 2000 extant nodes without altering the underlying simulation (**Figure 6**).

### Translational application of the hybrid OU–Branching framework

An OU–Branching simulator was developed in Python 3.11 to model therapy-modulated phenotypic and clonal dynamics under the *priA* profile. OU parameters were taken from replicate-grouped empirical fits on the log_10_ mutation-frequency scale (**Table 1**; *μ* = −5.000, *θ* = 0.117, *σ* = 0.436) and coupled to a pharmacokinetic exposure function *C(t)* representing six 21-day treatment cycles.

Phenotypic evolution followed an OU stochastic differential equation, whereas population growth and extinction were modeled using a phenotype- and drug-dependent branching process (24). Division and death rates were defined as $\lambda (Y_{t},\;C)=\;\lambda_{0}\cdot exp(\alpha \cdot (Y_{t}-\mu_{0})-k\lambda \cdot g(C))$ and $\delta (Y_{t},\;C)=\;\delta_{0}\cdot exp(-\beta \cdot (Y_{t}-\mu_{0})+k\delta \cdot g(C))$, where g(C)=C/(C+EC50) describes drug saturation (25). Here, *μ*_0_ denotes the baseline (pre-therapy) optimum; in this illustrative implementation, we set *μ*_0_ = *μ* from the replicate-grouped OU fit. In this formulation, increasing *C* suppresses division and increases death, capturing therapy-induced growth inhibition on a scale-invariant phenotype axis.

Each regimen comprised 256 stochastic replicates simulated for 200 days with *Δt* = 0.5 day, encompassing six dosing cycles and post-treatment periods. The ensemble distributions of $Y_{t}$ and $N_{t}$ describe therapy-driven phenotypic convergence, oscillatory tumor suppression, and extinction probabilities in the *priA*-mutant background, demonstrating the translational potential of the hybrid OU–Branching model for pediatric precision oncology (**Figure 7**).

## Results

### Mutation frequency trajectories and Ornstein–Uhlenbeck model fitting across bacterial lineages

Lineage-specific mutational dynamics were quantified in three *E. coli* strains—wild-type (WT), *priA*, and *recG*—using a hybrid OU–Branching framework. Mutation-frequency trajectories (**Figure 2**) showed marked divergence among lineages. The *priA* mutants accumulated mutations at a higher rate, consistent with replication restart defects leading to hypermutability. In contrast, *recG* displayed reduced mutation frequency; despite large diffusion (*σ*), its strong mean reversion (*θ*) constrained long-run dispersion, consistent with a collapse-prone regime.

OU parameter estimates (**Figure 2**; **Table 1**) revealed significant heterogeneity across lineages. The equilibrium mean (*μ*) reflects the steady-state mutation frequency; on the original scale (10^*μ*), *priA* exceeded WT by ~60-fold (~1.8 orders of magnitude). The diffusion scale (*σ*) and stationary variance ( $\sigma^{2}$) also differentiated lineages, indicating more substantial random fluctuations in *priA*. The likelihood ratio test (2ΔlogL = 33.18, df = 6, *p* = 9.7 x 10^-6^) confirmed that lineage-specific OU parameters provided a significantly better fit than a shared-parameter null model (**Table 2**). Collectively, these results demonstrate that bacterial replication–repair mutants evolve under distinct stabilizing selection regimes that govern mutation accumulation.

### OU model fits and 95% envelopes for mutation-frequency dynamics in bacterial lineages

Model-based predictive accuracy was evaluated by comparing observed mutation-frequency data with OU model simulations using lineage-specific parameter estimates. The fitted trajectories (**Figure 3**) recapitulated empirical trends, with the *priA* mutant exhibiting higher mean mutation frequencies and broader variance envelopes than either WT or *recG*. The expanded 95% predictive interval for *priA* indicates increased stochastic drift and reduced phenotypic stability resulting from defective replication restart pathways.

In contrast, WT and *recG* lineages displayed tightly constrained envelopes centered around low equilibrium values, reflecting stronger stabilizing selection and minimal stochastic deviation. Across replicate simulations, 95% of observed data points fell within the OU-predicted intervals, validating the robustness of the lineage-specific maximum-likelihood parameterization. Collectively, these model fits reveal distinct selective regimes influencing mutation-frequency evolution across the three bacterial genetic backgrounds.

### Uncertainty quantification of OU parameter estimates

To quantify uncertainty beyond point estimates and profile-likelihood geometry, we performed replicate-bootstrap refitting of the OU model (B = 2000). Across lineages, *μ, θ*, and *σ* showed lineage-specific uncertainty profiles and evident *θ*–*σ* coupling (**Table 1**). In particular, *priA* exhibited a low mean-reversion rate (*θ* ≈ 0.117) with strong *θ*–*σ* coupling (corr[log*θ*, log*σ*] ≈ 0.993), yielding the largest stationary variance ( $\sigma^{2}$/*(2θ)* median ≈ 0.817, 95% interval ≈ [0.753, 0.909]) on the log_10_ scale.

### Quantitative trajectory separation and sensitivity to parameter uncertainty

We complemented predictive envelopes with formal pairwise trajectory-separation metrics based on OU predictive distributions (**Table 3**), propagating uncertainty using replicate-grouped bootstrap parameter draws from **Table 1**. Across time points, *priA* showed strong separation from *recG* (D_mean ≈ 1.944 log10 units, 95% interval [1.912, 1.987], corresponding to ≈ 87.8-fold separation) and from WT (D_mean ≈ 1.045 log_10_ units, 95% interval [1.018, 1.091], corresponding to ≈ 11.1-fold separation). The *recG*–WT comparison showed smaller divergence (D_mean ≈ 0.898 log_10_ units, 95% interval [0.881, 0.917]). Bootstrap intervals were narrow relative to effect sizes, indicating robust qualitative regime separation.

### Profile-likelihood analysis of OU parameters (*θ*, *σ*) for *E. coli* lineages

Two-dimensional profile-likelihood surfaces were computed for the mean-reversion rate (*θ*) and diffusion scale (*σ*) across *E. coli* lineages (**Figures 4A–C**). The WT and *recG* surfaces exhibited narrow elliptical contours centered on distinct optima, indicating well-constrained parameter estimates. In contrast, the *priA* mutant displayed a broad and skewed likelihood surface, reflecting strong coupling between *θ* and *σ* and greater stochastic uncertainty. The shallow curvature of the *priA* ΔNLL landscape suggests that multiple parameter combinations produce comparable likelihoods, consistent with higher intrinsic noise and reduced stabilizing selection in its evolutionary dynamics.

Fit summaries (NLL and AIC) are reported for reference (**Table 2**). Consistent with the profile-likelihood geometry, *priA* shows strong *θ*–*σ* coupling and broader uncertainty, whereas WT and *recG* exhibit more sharply constrained optima (**Figure 4**).

### Coupled phenotypic and population-dynamics phase-planes

Phase-plane diagrams derived from stochastic simulations of the hybrid OU–Branching model captured the dynamic interplay between phenotypic evolution ( $Y_{t}$) and population size ( $N_{t}$, log scale). Each *E. coli* lineage exhibited a distinct coupling pattern between OU-driven phenotypic trajectories and branching-process demography.

The WT lineage exhibited smooth, monotonic trajectories, characterized by a gradual, well-regulated reduction in population size, accompanied by phenotypic reversion toward the lineage mean (*μ*). This behavior reflects strong stabilizing selection and minimal diffusion scale (*σ*), consistent with robust replication fidelity and steady population maintenance (**Figure 5A**). In contrast, the *priA* mutant showed large-amplitude oscillations in $Y_{t}$ and broad fluctuations in $N_{t}$ spanning several orders of magnitude, indicating mutator-driven stochastic drift and weakened phenotypic constraint (**Figure 5B**). The *recG* lineage remained confined near the equilibrium boundary, exhibiting modest noise and limited recovery following population collapse, which highlights impaired adaptive flexibility under replication-fork repair deficiency (**Figure 5C**).

The overlaid vector fields visualize the joint OU–Branching dynamics, with horizontal components representing phenotypic reversion toward *μ* and vertical components indicating exponential growth–decay cycles driven by the branching process. The blue trajectories trace reversion phases associated with population decline, most pronounced in WT, where stabilizing selection suppresses stochastic noise. Together, these results demonstrate that the hybrid OU–Branching framework accurately captures lineage-specific evolutionary equilibria, transient instability, and the contrasting balance between stabilizing selection and stochastic drift in DNA-repair-deficient lineages.

### Hybrid OU–Branching lineage architectures across *E. coli* lineages

Hybrid OU–Branching simulations generated lineage networks that visualized the balance between stabilizing selection and stochastic diversification across DNA-repair backgrounds.

In the WT lineage, network connectivity remained compact, with few extant subclones and narrow phenotypic dispersion around the equilibrium mean, consistent with strong stabilizing selection and low diffusion-driven variability (**Figure 6A**). The *priA* lineage exhibited extensive branching, higher node density, and broader color gradients, reflecting increased phenotypic drift and elevated mutation rate (**Figure 6B**). In contrast, the *recG* lineage produced sparse and short-lived branches, consistent with impaired replication restart and reduced adaptive potential (**Figure 6C**). Despite high *θ*, *recG* lineage underwent rapid lineage extinction and minimal diversification, highlighting the interplay between deterministic constraint and stochastic collapse. Collectively, these results demonstrate that OU–Branching coupling produces lineage architectures that distinguish stable (WT), hypermutable (*priA*), and collapse-prone (*recG*) evolutionary modes.

### Hybrid OU–Branching dynamics under therapy

To illustrate how the OU–Branching formalism can serve as a forward-simulation testbed for therapy-modulated evolution, we performed in silico simulations using the *priA* profile parameters inferred from replicate-grouped OU fits (**Table 1**; *μ* = −5.000, *θ* = 0.117, *σ* = 0.436) (**Figure 7**). These simulations are illustrative and hypothesis-generating: OU parameters are held fixed, drug exposure enters phenomenologically through the birth–death rates, and the results are not calibrated to patient response data or intended as prescriptive scheduling recommendations.

Panel A shows phenotypic trajectories ( $Y_{t}$, blue) and drug exposure *C(t)* (red) across six 21-day treatment cycles. Under this parameterization, phenotypes oscillate with dosing intervals and, on average, relax toward the long-run optimum *μ*, illustrating how cyclic exposure can interact with mean reversion (*θ*) and diffusion (*σ*) to shape temporal trait variability.

Panel B displays simulated population dynamics ( $N_{t}$) on a logarithmic scale. Treated simulations (red) exhibit oscillatory suppression and rebound before approaching a lower plateau relative to untreated controls (gray dashed), reflecting repeated perturbations of the division and death rates $\lambda (Y_{t},\;C)$ and $\delta (Y_{t},\;C)$ that modulate net growth.

Panel C shows an example OU–Branching lineage architecture at *t* = 20 days, with clones colored by phenotype ( $Y_{t}$) and edges scaled by the instantaneous division rate $\lambda (Y_{t},\;C)$. The topology illustrates early diversification followed by pruning of peripheral branches under phenotype-coupled demography.

Panel D presents an illustrative cure-probability map *P(cure)* as a function of diffusion (*σ*) and selection strength (*θ*). Higher *θ* and lower *σ* favor extinction, whereas weaker mean reversion or higher stochastic diffusion promotes persistence/relapse.

Together, these simulations demonstrate—in an illustrative, non-predictive setting—how coupling OU-based phenotypic drift with branching process dynamics can generate therapy-associated stabilization, clonal pruning, and extinction-versus-persistence regimes that motivate future calibration on clinical longitudinal measurements.

## Discussion

The hybrid Ornstein–Uhlenbeck (OU)–Branching framework offers a quantitative way to reason about therapy-associated dynamics and persistence by jointly modeling (i) therapy-shaped fluctuations of a latent or measured phenotypic state $Y_{t}$ and (ii) stochastic clonal survival through birth–death demography. Using *priA*-profile parameters inferred from replicate-grouped OU fits (*μ* = −5.000, *θ* = 0.117, *σ* = 0.436; **Table 1**) as a calibrated high-plasticity reference, we implemented an illustrative in silico cyclic-therapy regimen in which drug exposure modulates division and death rates while the trait dynamics remain mean-reverting (**Figures 7A, B**). In this setting, repeated dosing narrows phenotypic dispersion and induces oscillatory suppression–rebound dynamics in tumor burden as peripheral subclones are pruned during treatment and re-expand between cycles. Critically, $Y_{t}$ should be interpreted as an abstract phenotype axis in these simulations; in real tumors, it can be instantiated using longitudinal observables such as drug tolerance or differentiation-state scores from serial single-cell profiling, MRD trajectories, or clonal fraction proxies from time-series sequencing. This provides a direct path to patient-specific use: estimate (*μ*, *θ*, *σ*) from repeated measurements, couple them to therapy-dependent birth–death terms (and, where appropriate, therapy-dependent shifts in *μ*/*θ*/*σ*), and then explore how small changes in stabilizing strength or stochasticity alter extinction versus persistence regimes.

### Linking microbial and tumor evolutionary dynamics

The *E. coli* LTEE provides a tractable analog for studying general principles of tumor evolution, enabling direct observation of clonal diversification, stabilizing selection, and extinction events over thousands of generations (14, 26–28). Analyses of *priA* and *recG* mutants revealed lineage-specific differences in mutation frequency, phenotypic stability, and adaptive potential. The *priA* lineage displayed high stochastic variance and weak stabilizing selection, consistent with a mutator phenotype that parallels hypermutable pediatric tumor subclones. Conversely, *recG* exhibited constrained diversification and frequent extinction, reminiscent of repair-deficient cancer lineages with limited adaptive flexibility. These distinct evolutionary regimes—stabilized, hypermutable, and collapse-prone—represent fundamental modes of tumor evolutionary behavior within a shared mathematical landscape.

### Integrating stochasticity and selection through OU–Branching coupling

Classical models of cancer evolution often separate selection-driven deterministic growth from stochastic noise resulting from mutation or genetic drift (29–31). The hybrid OU–Branching model unifies these forces, allowing stochastic fluctuations to coexist with restoring selection toward a phenotypic optimum. The OU process captures trait-level stability and noise, whereas the branching component introduces demographic uncertainty via random birth–death processes (32–35). This integration explains how even low-mutation pediatric tumors can exhibit high intratumoral heterogeneity: stochastic phenotypic variation is continually generated and pruned by stabilizing selection, thereby maintaining evolutionary plasticity without requiring substantial mutational input.

### Quantitative insights into lineage evolution

Parameter inference and simulation analyses provide quantitative evidence that stochastic variance (*σ²*) and mean-reversion strength (*θ*) determine lineage diversification and persistence. Lineages characterized by high *σ²* and low *θ*, such as *priA*, exhibit broad phenotypic dispersion and long-tailed lineage survival distributions. In contrast, lineages with strong mean reversion (high *θ*)—including WT and *recG*—exhibit constrained long-run dispersion (*σ²*/(*2θ*)) and reduced persistence of extreme phenotypic excursions. The model formalizes an evolutionary trade-off between plasticity and stability: excessive stochasticity promotes transient adaptation but risks instability, whereas strong reversion constrains exploration while preserving lineage integrity. This trade-off may underlie the tendency of some pediatric cancers to relapse following therapy, as treatment pressures perturb *θ* and *σ²*, altering the effective “evolutionary temperature” of the tumor ecosystem. These regime distinctions are supported by trajectory-separation metrics (**Table 3**), with *priA* showing the strongest separation from *recG* and WT.

### Translational implications for therapy modeling

The hybrid OU–Branching framework provides a unifying stochastic language for therapy-modulated evolution by coupling mean-reverting phenotypic dynamics with demographic birth–death branching. Here, we use therapy simulations as an illustrative forward-simulation testbed to show how the model behaves under cyclic exposure when OU parameters are held fixed, and treatment effects are introduced phenomenologically through exposure-modulated birth and death rates. Accordingly, these simulations are hypothesis-generating and are not calibrated to clinical response data, nor do they imply optimal scheduling or clinical efficacy.

Using the *priA* profile parameters inferred from replicate-grouped OU fits (*μ* = −5.000, *θ* = 0.117, *σ* = 0.436; **Table 1**), the model generates cyclic oscillations of phenotype ( $Y_{t}$) and population size ( $N_{t}$) across six 21-day exposure cycles (**Figures 7A, B**). Under this parameterization, phenotypic trajectories fluctuate with dosing intervals and, on average, relax toward the long-run optimum *μ*, illustrating how the balance between mean reversion (*θ*) and stochastic diffusion (*σ*) can shape therapy-associated variability over time. In parallel, population trajectories exhibit a suppression–rebound pattern under repeated exposure, consistent with alternating periods of demographic contraction and regrowth in the branching process.

When the same fixed OU parameters are used to generate lineage networks at *t* = 20 days (**Figure 7C**), the resulting architecture shows early diversification followed by extinction of peripheral branches, reflecting stochastic pruning that can arise when demographic rates depend on the evolving phenotypic state.

The outcome surface in **Figure 7D** is presented as an illustrative regime diagram: within the model, higher *θ* and lower *σ* shift simulations toward extinction, whereas weaker mean reversion or larger stochastic diffusion favors persistence. We emphasize that *P(cure)* here denotes a model-based extinction probability under the assumed dynamics, rather than a clinically validated cure probability.

In summary, the therapy simulations demonstrate—in a deliberately simplified, non-predictive setting—how coupling OU-based phenotypic drift with branching-process demography can produce oscillatory trajectories, clonal pruning, and extinction-versus-persistence regimes. A key next step is to calibrate $Y_{t}$ to longitudinal tumor measurements (e.g., MRD/VAF-derived burden proxies, inferred drug-tolerance or stress-response programs from serial single-cell profiling, or pathway activity scores) and to estimate patient- or cohort-specific parameters. Such calibration would enable principled sensitivity analyses and comparative testing of evolution-aware hypotheses across therapeutic contexts, without implying prescriptive optimization or clinical prediction in the present study.

### Model limitations and future directions

Although the hybrid OU–Branching framework captures foundational evolutionary principles, several important limitations should be acknowledged. First, the current formulation reduces phenotypic evolution to a single latent trait evolving under effective stabilizing forces, whereas real tumors evolve on multidimensional, context-dependent fitness landscapes shaped by metabolism, epigenetic plasticity, microenvironmental heterogeneity, and therapy-induced state transitions. Extending the model to multi-trait (vector) OU dynamics, potentially with hierarchical coupling among traits and patient-specific covariates, would better reflect the polygenic and multi-axis nature of tumor adaptation.

Second, the present branching process is an effectively well-mixed, lineage-level approximation that does not explicitly encode spatial structure, local resource limitation, or niche partitioning. Spatial constraints, dispersal, and local competition can substantially alter clonal dynamics in tumors and generate distinct evolutionary modes (e.g., branching evolution, neutral expansion, or clonal sweeps) (36, 37). The well-mixed approximation is most reasonable when the modeled state variable and the data are lineage-aggregated (e.g., clonal fraction trajectories) and/or in regimes where mixing is enhanced, and population sizes are relatively small—such as minimal residual disease (MRD) or post-treatment residual disease in hematologic malignancies—but it is less appropriate for strongly structured solid tumors. Incorporating density dependence (carrying capacity), spatial embedding, and explicit niche structure would broaden biological realism and improve applicability across tumor types.

Relatedly, recent advances in spatial genomics and clone-mapping techniques (e.g., base-specific *in situ* sequencing) enable reconstruction of spatial clonal topologies and mutational landscapes in human tumors (38). These data provide a direct opportunity to align model behavior with spatially resolved lineage structure. Experimental validation remains critical: in pediatric leukemia or solid tumors, serial measurements such as time-series variant allele fractions, MRD-linked burden proxies, and (where feasible) single-cell lineage tracing could be used to estimate OU–Branching parameters (*θ*, *σ²*) under an explicit observation-noise model. Perturbation experiments targeting DNA repair, replication stress, or chromatin regulation could then test whether inferred parameter shifts are associated with increased lineage extinction versus persistence.

In future work, the model could be expanded to incorporate multiple traits, spatial structure, microenvironmental niches, and cell–cell interactions. Embedding OU–Branching dynamics within spatial frameworks such as the Cellular Potts Model or lattice-based spatial automata (e.g., BIO-LGCA) may help explore how migration, adhesion, and heterogeneity shape evolutionary trajectories (39–42). Finally, close collaboration with longitudinal oncology studies that collect repeated measurements across therapy will be essential to calibrate and validate the framework in real tumor systems.

For pediatric tumors, the OU–Branching state variable $Y_{t}$ can be instantiated as a longitudinally measurable cell-state or burden proxy, rather than mutation frequency per se. Practical choices include: (i) drug-tolerance or stress-response scores and differentiation-state programs derived from serial single-cell RNA-seq; (ii) MRD-linked burden metrics or log-transformed tumor fraction trajectories; and (iii) clonal fraction proxies from time-series sequencing (e.g., VAF- or clone-abundance–based summaries). With $Y_{t}$ defined and repeatedly measured, patient- or cohort-specific (*μ*, *θ*, *σ*) can be estimated using an observation model that accounts for measurement noise and sampling variability, enabling calibrated forward simulations that connect stabilization versus plasticity to lineage persistence and treatment sensitivity.

## Conclusions

The hybrid OU–Branching framework provides a quantitative bridge between evolutionary biology and translational oncology by linking constrained (mean-reverting) phenotypic dynamics with stochastic lineage diversification and extinction. By uniting microbial and cancer evolution within a shared stochastic–stabilizing paradigm, it offers a principled way to interpret how pediatric tumors may retain adaptive flexibility despite low mutation burdens, while remaining subject to effective stabilizing forces.

More broadly, the key contribution of this work is a generalizable inferential formalism that couples continuous trait evolution and discrete branching within a single likelihood-based framework. This unified perspective enables coherent estimation of latent trajectories and lineage architecture from longitudinal data, and it provides a transparent foundation for future calibration to clinical time-series measurements to support evolution-aware hypothesis testing in pediatric oncology.

## Data Availability

All data analyzed in this study are included in the article and its Supplementary Material. Code used for analyses and figure generation is publicly available in a GitHub repository: https://github.com/shkim9391/hybrid-OU-branching-pediatric-evolution.
