# Supplementary material for: A hybrid Ornstein–Uhlenbeck–Branching framework unifies microbial and pediatric tumor evolution
Source: Front Oncol. 2026 Feb 13;16:1727973. doi: 10.3389/fonc.2026.1727973 (PMC12954049; doi:10.3389/fonc.2026.1727973)
Supplement: Supplementary file 1 [file DataSheet1.pdf]

Supplementary Figure S1. Lifespan and time-horizon comparison: bacterial vs. pediatric cancer evolution

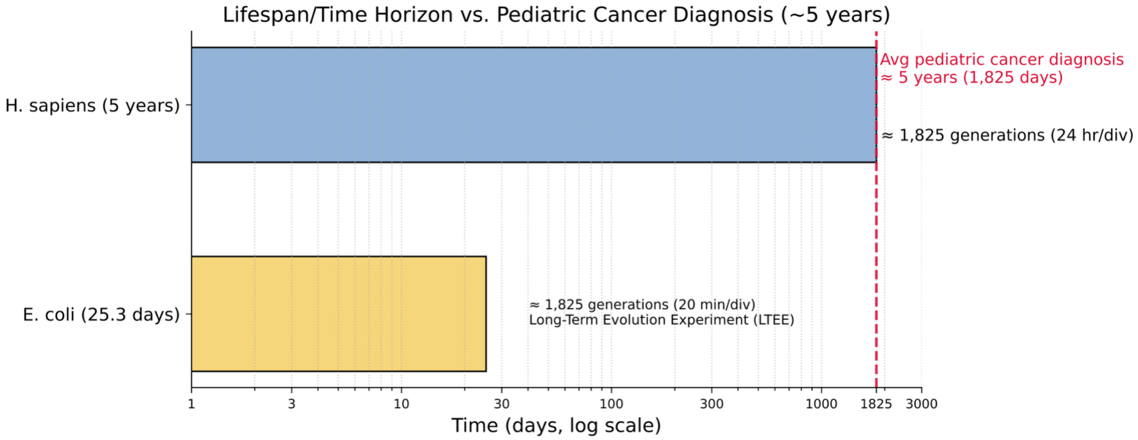

Schematic comparison of characteristic time horizons in microbial evolution versus pediatric cancer. For bacteria, we report a division-equivalent horizon based on a representative doubling time of ~20 minutes in rich medium (≈72 divisions/day), so a ~25-day experimental window corresponds to ≈1,800 cell divisions (division equivalents). For pediatric cancers, we summarize clinically relevant developmental/diagnostic time scales (months to years). This figure is intended as an order-of-magnitude comparison of evolutionary opportunity rather than a strict mapping of experimental protocols.

Supplementary Figure S2. Experimental framework of a serial-transfer *E. coli* evolution experiment (division-equivalent view)

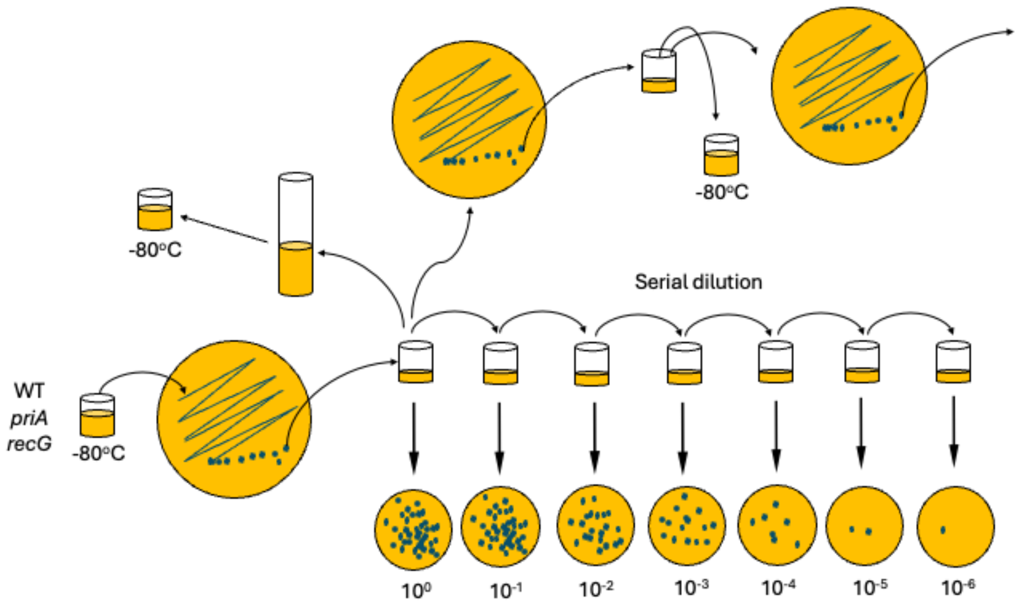

Schematic of a serial-transfer design with daily dilution/transfer cycles. To contextualize evolutionary opportunity on a common scale, we annotate a division-equivalent rate assuming a ~20-minute doubling time in rich medium (≈72 divisions/day). This framing is used only to express an approximate division-equivalent time horizon; inference in the main text uses the observed sampling time points and does not rely on a specific generations-per-day assumption.

Supplementary Table S1. Raw Mutation Frequency Data

Lineage	Replicate	Day	Mutation Frequency
WT	1	3	1.33E-07
WT	1	6	8.89E-08
WT	1	9	0
WT	1	12	0
WT	1	15	2.67E-07
WT	1	18	1.11E-07
WT	1	21	2.51E-06
WT	1	24	1.78E-07
WT	2	3	1.14E-07
WT	2	6	8.50E-08
WT	2	9	0
WT	2	12	0
WT	2	15	2.57E-07
WT	2	18	1.09E-07
WT	2	21	2.75E-06
WT	2	24	1.89E-07
WT	3	3	1.30E-07
WT	3	6	8.80E-08
WT	3	9	0
WT	3	12	0
WT	3	15	2.66E-07
WT	3	18	1.25E-07
WT	3	21	3.48E-06
WT	3	24	1.69E-07
priA	1	3	0
priA	1	6	0
priA	1	9	4.00E-07
priA	1	12	4.67E-07
priA	1	15	4.44E-07
priA	1	18	3.22E-05
priA	1	21	1.58E-05
priA	1	24	1.58E-05
priA	2	3	0
priA	2	6	0
priA	2	9	9.00E-07
priA	2	12	5.67E-07

priA	2	15	5.49E-07
priA	2	18	4.97E-05
priA	2	21	1.74E-05
priA	2	24	1.37E-05
priA	3	3	0
priA	3	6	0
priA	3	9	3.00E-07
priA	3	12	4.55E-07
priA	3	15	4.55E-07
priA	3	18	2.78E-05
priA	3	21	1.47E-05
priA	3	24	1.79E-05
recG	1	3	0
recG	1	6	0
recG	1	9	2.22E-08
recG	1	12	0
recG	1	15	0
recG	1	18	1.11E-06
recG	1	21	0
recG	1	24	0
recG	2	3	0
recG	2	6	0
recG	2	9	2.45E-08
recG	2	12	0
recG	2	15	0
recG	2	18	1.10E-06
recG	2	21	0
recG	2	24	0
recG	3	3	0
recG	3	6	0
recG	3	9	2.10E-08
recG	3	12	0
recG	3	15	0
recG	3	18	7.00E-07
recG	3	21	0
recG	3	24	0

Mutation-frequency trajectories used for Ornstein–Uhlenbeck (OU) parameter estimation (μ, θ, σ) in the main text (Figures 2 and 3). Each lineage was sampled at 8 time points (Day

3–24) with three independent replicates. Values represent mutation frequencies (fraction of mutant alleles per total population). Zeros denote undetected mutations at the assay detection limit ($\sim 10^{-8}$).

Supplementary Table S2. Fit summary (NLL and AIC) for replicate-grouped OU fits by lineage.

**Lineage**	**NLL**	**AIC**	**ΔAIC**
**WT**	18.388	42.777	0
priA	20.463	46.925	4.149
recG	21.572	49.145	6.368

For each lineage (WT, *priA*, *recG*), OU parameters (μ, θ, σ) were estimated by maximum likelihood using the exact OU transition density, with the negative log-likelihood (NLL) summed across replicate trajectories (replicate-grouped likelihood). AIC was computed as AIC = 2k + 2×NLL with k=3 parameters per lineage-specific OU fit. ΔAIC is reported relative to the smallest AIC in this table as a descriptive reference.
